# Prognostic Implication of the m^6^A RNA Methylation Regulators in Rectal Cancer

**DOI:** 10.3389/fgene.2021.604229

**Published:** 2021-06-03

**Authors:** Yajie Chen, Shanshan Wang, William C. Cho, Xiang Zhou, Zhen Zhang

**Affiliations:** ^1^Department of Radiation Oncology, Fudan University Shanghai Cancer Center, Fudan University, Shanghai, China; ^2^Department of Oncology, Shanghai Medical College, Fudan University, Shanghai, China; ^3^Fudan University Shanghai Cancer Center and Institutes of Biomedical Sciences, Fudan University, Shanghai, China; ^4^Department of Clinical Oncology, Queen Elizabeth Hospital, Hong Kong, China; ^5^Key Laboratory of Breast Cancer in Shanghai, Fudan University Shanghai Cancer Center, Fudan University, Shanghai, China

**Keywords:** N6-methyladenosine, YTHDC2, METTL14, rectal cancer, prognosis

## Abstract

N6-methyladenosine (m^6^A) is a very common and abundant RNA modifications occurring in nearly all types of RNAs. Although the dysregulated expression of m^6^A regulators is implicated in cancer progression, our understanding of the prognostic value of the m^6^A regulators in rectal cancer is still quite limited. In this study, we analyzed the RNA expression levels of the 17 m^6^A regulator genes of 95 rectal cancer and 10 normal rectal samples from the The Cancer Genome Atlas Rectum Adenocarcinoma (TCGA-READ) dataset. Lasso regression analysis was conducted to build a prognostic model and calculate the risk score. The rectal cancer patients were then devided into the high-risk and low-risk groups according to the mean risk score. The prognostic value of the identified model was separately evaluated in the TCGA-READ and GSE87211 datasets. GSEA was conducted to analyze the functional difference of high-risk and low-risk rectal cancer patients. Our analysis revealed that rectal cancer patients with lower expression of YTHDC2 and METTL14 had a remarkable worse overall survival (*P* < 0.05). The prognostic value of the model was validated in GSE87211 datasets, with AUC = 0.612 for OS and AUC = 0.651 for RFS. Furthermore, the m^6^A modification-based risk score system is associated with activation of distinct signaling pathways, such as DNA repair, epithelial-mesenchymal transition, G_2_M checkpoint and the MYC pathway, that may contribute to the progression of rectal cancer. In conclusion, our findings demonstrated that the m^6^A RNA methylation regulators, specifically YTHDC2 and METTL14, were significantly down-regulated and might be potential prognostic biomarkers in rectal cancer.

## Introduction

Rectal cancer (RC) is one of the most common malignant tumors in the digestive system with high incidence and mortality, bringing great challenges to human health (Chen et al., 2016; Siegel et al., 2020). Surgery is the only curable treatment for early RC cases, preoperative chemoradiotherapy has become the standard treatment for the locally advanced RC (Benson et al., 2018; Heald and Ryall, 1986; Rodel et al., 2015). Over the past 30 years, mortality has decreased significantly in the wake of widespread colonoscopy uptake, but the survival rate for patients with advanced RC remains low (Allemani et al., 2018; Siegel et al., 2020). Hence, dissecting the molecular mechanism of RC pathogenesis and identifying novel prognostic biomarkers could be beneficial to the diagnosis and treatment of RC patients.

Post-transcriptional modifications have emerged as important regulators in cancer initiation and progression, and attracted increasing attention in cancer research (Barbieri and Kouzarides, 2020). Thus far, more than 100 different types of post-transcriptional modifications of RNA have been identified in all living organisms according to the MODOMICS database (Boccaletto et al., 2018). N6-methyladenosine (m^6^A), the methylation at the N6 position of adenosine, has proved to be the most common, abundant and conserved modification found in nearly all types of RNAs (Desrosiers et al., 1974; Fazi and Fatica, 2019). The m^6^A modification is highly enriched around the stop codon area, 3’ untranslated region (UTR), and within the coding region, thereby playing an essential role in RNA turnover, translation, and other processes (Deng et al., 2018; He et al., 2019; Meyer et al., 2012). The m^6^A modification involves three types of critical molecules, methyltransferases, demethylases, and m^6^A binding proteins (Zaccara et al., 2019). Specifically, m^6^A methylation is catalyzed by the methyltransferases (termed as “writers”), including METTL3, METTL14, ZC3H13, RBM15, KIAA1429, and WTAP, and reverted by the demethylases (termed as “erasers”), such as FTO and ALKBH5. The m^6^A binding proteins (termed as “readers”), including YTHDC1, YTHDC2, YTHDF1, YTHDF2, YTHDF3, IGF2BP1, IGF2BP2, IGF2BP3, and HNRNPC, are responsible for mediating different actions of the m^6^A modification that may lead to diverse cellular outcomes (Panneerdoss et al., 2018).

Recently, accumulating evidence has suggested that the dysregulated expression of m^6^A RNA methylation regulators is strongly involved in cancer progression (Lan et al., 2019). For instance, the m^6^A methyltransferase METTLE3 was overexpressed in human lung cancer, liver cancer, and acute myeloid leukemia (AML) as an oncogenic protein during cancer development (Barbieri et al., 2017; Lin et al., 2016; Lin et al., 2019). The demethylase FTO was also found to promote progression of AML and breast cancer by preventing m^6^A modification from the target mRNAs (Li et al., 2017; Niu et al., 2019). The m^6^A reader protein IGF2BP1 could activate SRF-dependent transcription and thus endorse tumor cell growth in an m^6^A-dependent manner in ovarian, liver, and lung cancers (Muller et al., 2019). In colorectal cancer, the reader YTHDF1 was found to be overexpressed and associated with the stem-like features of cancer cells (Bai et al., 2019; Nishizawa et al., 2018). Taken together, the m^6^A regulators have been shown to modulate gene expression in a broad spectrum, and elucidating the molecular basis and clinical significance of m^6^A modification remain an active area of investigation in cancer.

Although several m^6^A-related genes has been implicated in prognosis of colorectal cancer (Liu T. et al., 2019), our understanding of the prognostic value and function of the m^6^A regulators in RC is still quite limited. Thus, we investigated the expression pattern of the m^6^A regulator genes in RC tissues and its correlation with RC prognosis based on the data from The Cancer Genome Atlas (TCGA) and Gene Expression Omnibus (GEO) in this study.

## Methods

### Data Source and Processing Method

Two independent datasets, The Cancer Genome Atlas Rectum Adenocarcinoma (TCGA-READ) and GSE87211, were analyzed for the construction and validation of the prognostic model. In the TCGA-READ dataset, the read count data of RNA sequence of a total of 95 RC patients and 10 normal adjacent tissues was downloaded. The Ensemble IDs of the 17 m^6^A methylation regulators (METTL3, METTL14, ZC3H13, RBM15, KIAA1429, WTAP, FTO, ALKBH5, YTHDC1, YTHDC2, YTHDF1, YTHDF2, YTHDF3, IGF2BP1, IGF2BP2, IGF2BP3 and HNRNPC) were translated into the official gene symbols according to the human reference genome assembly (version GRCh38). In addition, the information of patients’ clinical characteristics (gender, age, and tumor stage) and survival status [overall survival (OS) and relapse free survival (RFS)] was further obtained to generate a comprehensive matrix along with the gene expression information.

The GSE87211 dataset recorded the gene expression profiling data of 203 RC tumor tissue samples that was generated from the Agilent-026652 Whole Human Genome Microarray 4 × 44K v2 (GPL13497 platform). The expression levels of the 17 m^6^A methylation-related genes and follow-up information of the 203 RC patients were combined into another matrix for the external validation of the identified prognostic signature.

### Identification of the Survival-Related Signature

The least absolute shrinkage and selection (lasso) regression method using the selection operator algorithm as described (Liu et al., 2020; Meng et al., 2018; Wang et al., 2020) was applied to construct a prognostic model with the highest efficiency and least redundancy based on the expression levels of the 17 genes. The factors included in the optimal model were selected using the ‘glmnet’ [20808728] and ‘caret’ [Kuhn M, Wing J, Weston S, et al. Caret: Classification and Regression Training; 2016. https://CRAN.R-project.org/package=caret] R packages. The risk score of each patient was thus estimated by the identified model for further analysis.

### Assessment Method of the Prognostic Model

Based on the mean value of the estimated risk scores in the TCGA-READ dataset, all the RC patients were separated into high-risk and low-risk subgroups. The mean risk score of all samples was measured – a sample with lower risk score was considered the low-risk sample, otherwise, it was considered a high-risk sample. The survival status of the two subgroups was compared using the Kaplan-Meier curve survival analysis. A two-sided *P* value <0.05 was considered to be of significance. The predictive performance of the model was evaluated using the receiver operating characteristic (ROC) curve analysis. Univariate and multivariate Cox regression analyses were performed to estimate whether the calculated risk score was an independent prognostic factor for RC regardless of patients’ clinical characteristics. A nomogram integrating risk score and multiple clinicopathological risk factor was then constructed to predict the prognostic value. All the statistical analysis and figure formation processes in this study were performed using the R software (version 3.6.3).

### Gene Set Enrichment Analysis (GSEA)

The potential mechanisms involved by the identified signature were further explored by GSEA based on the expression profiles of all the protein-coded genes of RC patients from the TCGA-READ dataset. The biological difference of the high-risk and low-risk RC patients was compared using the GSEA software (version 4.1.0). The classical Kyoto Encyclopedia of Genes and Genomes (KEGG) pathway and Gene Ontology (GO) annotation assemblies were included for analysis. The false discovery rate (FDR) and normalized enrichment score (NES) was estimated for each pathway or process. A normalized *P* value <0.05 was considered to be significant.

## Results

### Expression of the m^6^A Methylation Regulator Genes in the TCGA-READ Dataset

**FIGURE 1:**
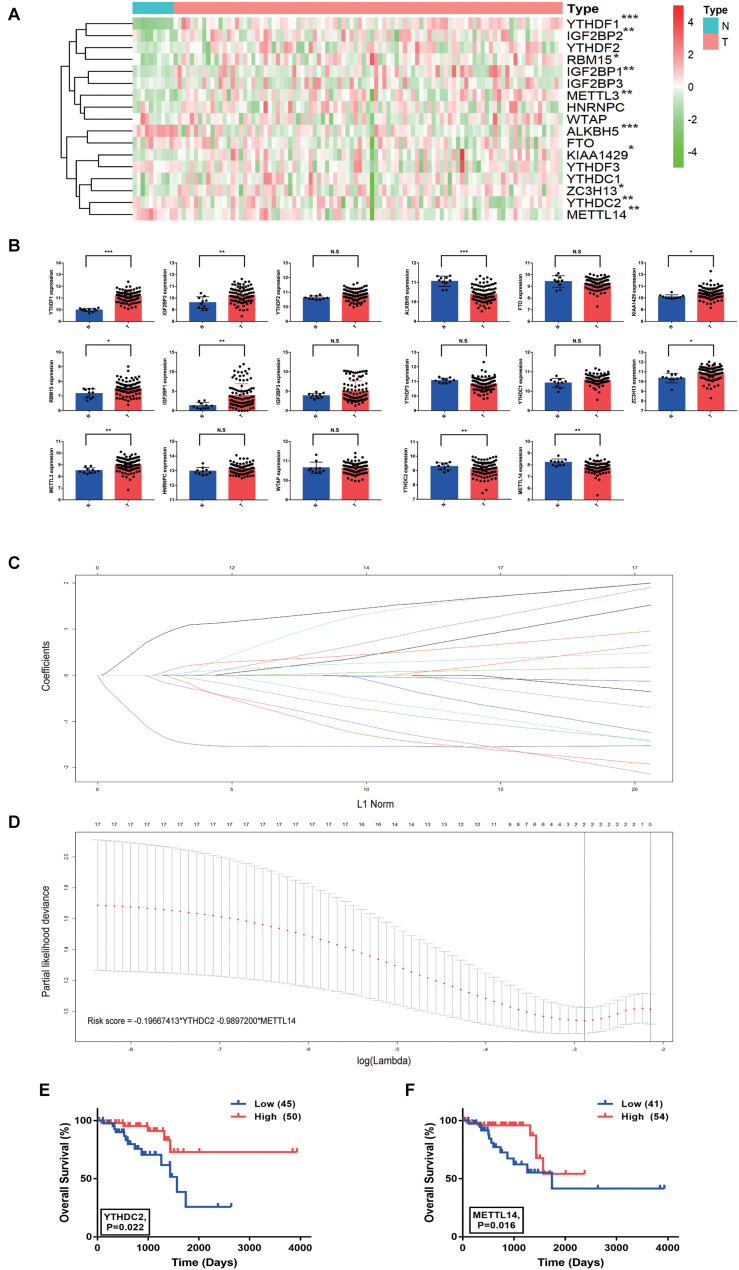
Risk signature with two m^6^A RNA methylation regulator genes in rectal cancer. **(A)** The heatmap of the expression profiles of the 17 m^6^A RNA methylation regulator genes in the TCGA-READ dataset (N = 10, T = 95). **(B)** The expression of the 17 m^6^A regulator genes in tumor and normal samples (unpaired T-tests). **(C,D)** The coefficients estimated by multivariate Cox regression via LASSO are presented. **(E)** Comparison of overall survival with different YTHDC2 expression. **(F)** Comparison of overall survival with different METTL14 expression. *P < 0.05, **P < 0.01, and ***P < 0.001.

The RNA expression levels of the 17 m^6^A methylation regulator genes were compared between the 95 cancerous and 10 normal tissue samples from the TCGA-READ dataset using the Mann-Whitney *U* test. Ten of the 17 genes showed abnormal expression in tumor tissues compared with the normal tissues, which indicated the important role of m^6^A methylation in cancer development (Figure 1A). Specifically, YTHDF1, IGF2BP2, KIAA1429, RBM15, IGF2BP1, ZC3H13, and METTL3 were significantly up-regulated in tumor tissues by unpaired T-tests (*P* < 0.05), while ALKBH5, YTHDC2, and METTL14 were significantly down-regulated (*P* < 0.01) (Figure 1B). In addition, the RNA expression levels of the 17 m^6^A regulator genes were also analyzed in the 6 paired of rectal tumors and normal rectal samples using paired T-tests. The significant up-regulation of YTHDF1, YTHDF2, and METTL3, and down-regulation of ALKBH5, YTHDC2, and METTL14 could be observed in the 6 rectal tumors (*P* < 0.05) (Supplementary Figure 1), indicating that these m^6^A regulators may be associated with progression of RC.

### The Prognostic Value of the m^6^A Methylation Regulators in Rectal Cancer

**FIGURE 2:**
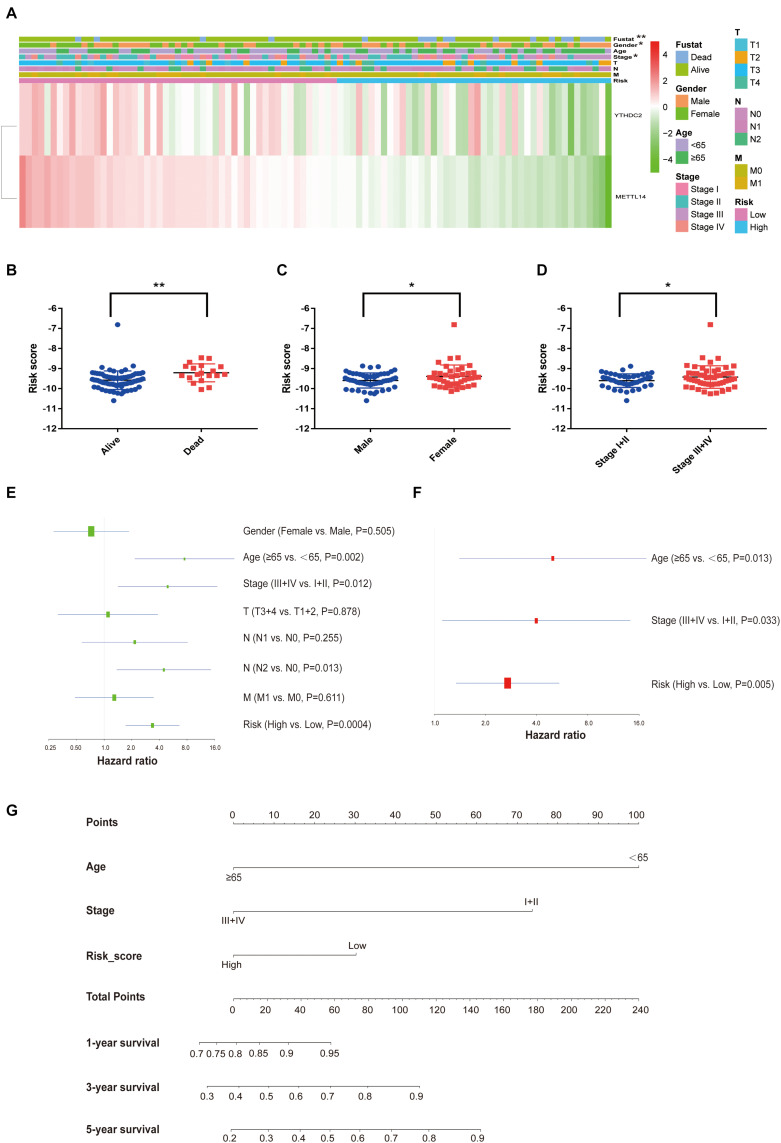
Relation between the risk score and clinicopathological characteristics in rectal cancer. **(A)** The heatmap shows the expression of the two m^6^A regulator genes in the high-risk and low-risk rectal cancer. **(B–D)** The distribution of risk score in patients stratified by the live status **(B)**, gender **(C)**, and pathological stage **(D)** (unpaired T-tests). **(E,F)** The forest plot of univariate and multivariate Cox regression analysis. **(G)** The nomogram integrating risk score and clinicopathological factors in the prediction model. *P < 0.05, **P < 0.01.

Lasso regression analysis was conducted to build a prognostic model based on the expression of m^6^A methylation regulators. The combination of two genes, YTHDC2 and METTL14, showed high potential in the risk prediction of RC patients (Figures 1C,D). In addition, the log-rank survival analysis suggested that the RC patients with lower expression of these two genes have a remarkable worse overall survival with *P* < 0.05 (Figures 1E,F). Additional m^6^A regulators, including HNRNPA2B1, RBM15B, RBMX, METTL16, FMR1, and LRPPRC, reported in a recent study (Zhang et al., 2020), were also analyzed (Supplementary Figure 2). The log-rank survival analysis suggested that the RC patients with lower expression of RBMX and LRPPRC have a remarkable worse overall survival with *P* = 0.012 and *P* = 0.003, respectively. However, unpaired T-tests results showed that RBMX and LRPPRC were significantly up-regulated in tumor tissues compared with normal tissues. Thus, these results excluded the 6 m^6^A regulators from our further analysis. For each patient, the risk score was calculated using the formula “-0.19667413 × YTHDC2 expression – 0.9897200 × METTL14 expression.” The relationship between the risk score and clinicopathological characteristics of RC patients was further explored in the TCGA-READ dataset (Figure 2A). It was observed that the dead (Figure 2B), female (Figure 2C), and late-stage (Figure 2D) patients have significantly higher risk scores than the corresponding controls. Based on the expression of YTHDC2 and METTL14, the univariate and multivariate Cox regression analyses revealed that the risk score can independently predict the survival of RC patients regardless of the varied clinical features with *P* = 0.0004 and *P* = 0.005, respectively (Figures 2E,F). A nomogram integrating the age, stage and risk score was built to estimate the prognosis of RC using the TCGA dataset (Figure 2G).

### The Prediction Efficiency of the Prognostic Model

**FIGURE 3:**
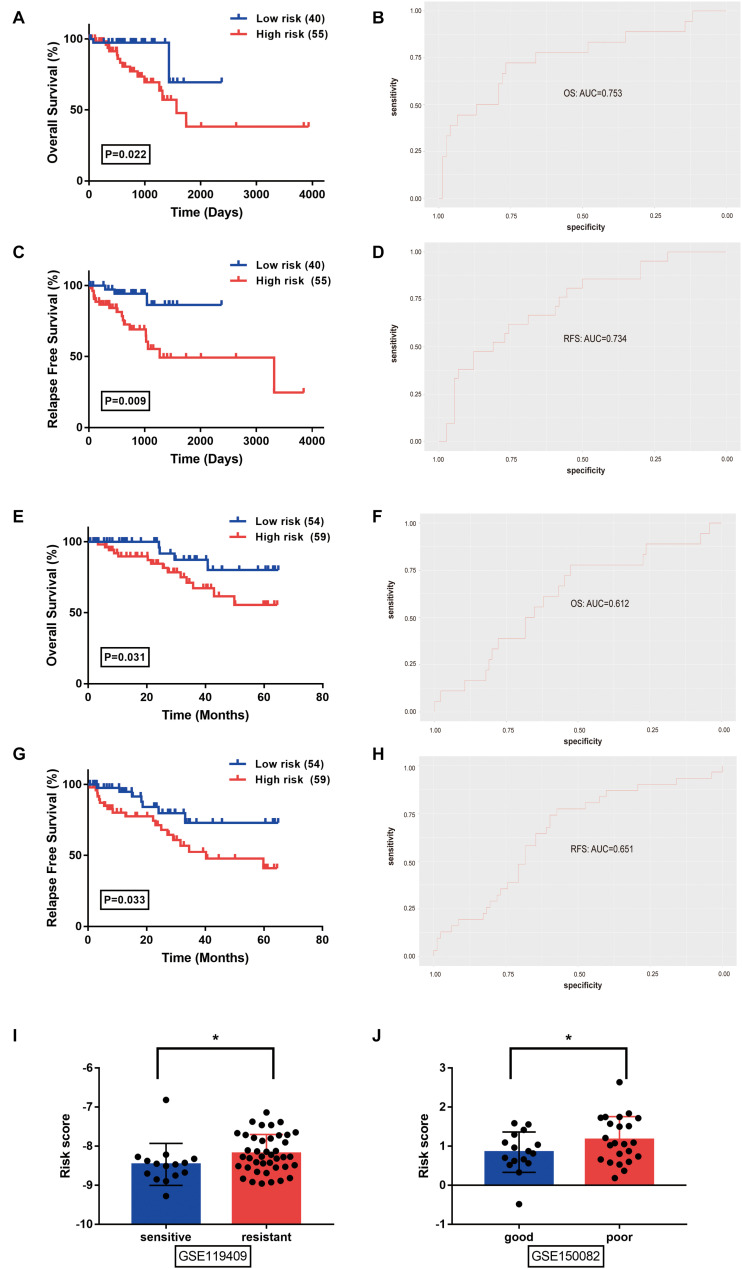
Prognostic value of the identified signature was evaluated in rectal cancer from the TCGA-READ **(A–D)**, GSE87211 **(E–H)**, GSE119409 **(I)**, and GSE150082 **(J)** datasets. **(A,E)** Overall survival analysis between low- and high- risk groups stratified by the risk score. **(B,F)** The receiver operating characteristic (ROC) curve was used to evaluate the predictive efficiency of the overall survival rate. **(C,G)** Relapse free survival analysis between low- and high- risk groups stratified by the risk score. **(D,H)** The ROC curve was used to evaluate the predictive efficiency of relapse free survival rate. **(I)** The distribution of risk score in rectal cancer patients stratified by the response to neoadjuvant radiotherapy (unpaired T-tests). **(J)** The distribution of risk score in locally advanced rectal cancer patients tratified by the response to chemoradiotherapy (unpaired T-tests). *P < 0.05.

The prognostic value of the identified model was separately evaluated in the TCGA-READ and GSE87211 (Figure 3) datasets. RC patients were divided into high- and low-risk subgroups according to the median value of the risk scores. In the TCGA-READ dataset, the results of Kaplan-Meier curves showed that the patients with lower risk scores have significantly longer OS and RFS periods than those with high scores (Figures 3A,C). The AUCs of the risk model to predict patients’ OS and RFS rates were 0.753 and 0.734, respectively (Figures 3B,D). The predictive performance of the model remained stable in the GSE87211 dataset. Patients in the high-risk group had a consistent worse overall prognosis and shorter RFS time compared with the low-risk group (Figures 3E,G), and the AUCs of the model to predict the OS and RFS rate of RC patients were 0.612 and 0.651, respectively (Figures 3F,H). Moreover, in the GSE119409 dataset, the results showed that the RC patients with no-response to neoadjuvant radiotherapy (*n* = 41) have significantly higher risk scores than the sensitive group (*n* = 15) (*P* < 0.05; Figure 3I). Analysis of the GSE150082 dataset revealed that locally advanced rectal cancer (LARC) patients with poor response to chemoradiotherapy (*n* = 23) have significantly higher risk scores than the patients with good response to neoadjuvant chemoradiotherapy treatment (nCRT) (*n* = 16) (*P* < 0.05; Figure 3J). Altogether, the identified m^6^A methylation-correlated signature showed stable efficiency in risk prediction for RC patients.

### Functional Enrichment Analysis

**FIGURE 4:**
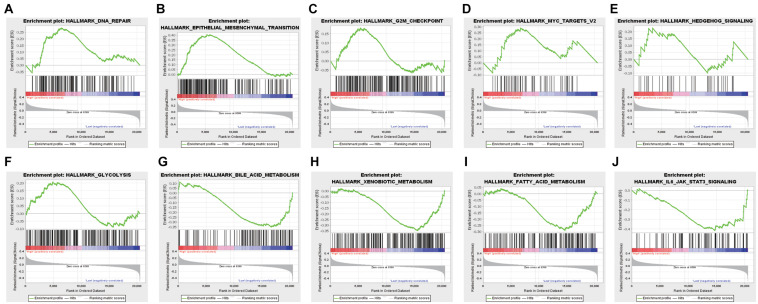
Genes or pathways associated with higher risk are enriched for hallmarks of malignant tumors: **(A)** DNA repair, **(B)** epithelial-mesenchymal transition, **(C)** G2M checkpoint, **(D)** Myc targets, **(E)** hedgehog signaling, **(F)** glycolysis, **(G)** bile acid metabolism, **(H)** xenobiotic metabolism, **(I)** fatty acid metabolism, and **(J)** JAK-STAT3 signaling.

GSEA was conducted to analyze the functional difference of high-risk and low-risk RC patients. Illustrated in Figure 4 are some of the most significant pathways or biological processes that were differentially enriched by the two groups. The high- and low-risk groups had distinct performance in certain cancer-related processes, including DNA repair, epithelial mesenchymal transition, G_2_M checkpoint, and the MYC pathway. Interestingly, several metabolism-related pathways, such as glycolysis and fatty acid metabolism, as well as the IL6-JAK-STAT3 pathway in immune response were also identified. Therefore, the m^6^A modification-based risk score system is not only able to predict clinical outcomes of RC patients, but also associated with activation of distinct signaling pathways that may contribute to the progression of RC.

## Discussion

RC, combined with colon cancer, ranks third in both the incidence of total tumor cases and is the cause of cancer-related death in the United States (Siegel et al., 2020). Although the survival trends for RC were generally flat during the past two decades, the 5-year survival rate of advanced patients remains at a relatively low level, making the proposal of appropriate prognostic models necessary for risk prevention (Allemani et al., 2018). Recently, studies have been done to describe the correlation of m^6^A RNA methylation regulators and cancer progression. Liu et al. investigated the prognostic value of 13 m^6^A RNA methylated regulatory factors in colon adenocarcinoma and identified YTHDF1 and HNRNPC as prognostic factors (Liu X. et al., 2019). Another study determined the expression patterns and prognostic value of 15 m^6^A-related genes in colorectal cancer (Liu T. et al., 2019). In this study, we analyzed a total of 17 m^6^A regulator genes in RC and demonstrated the prognostic value of YTHDC2 and METTL14 using the TCGA and GEO data.

The role and clinical significance of m^6^A modification in human cancers have gained increasing attentions in recent years, although it was first described in 1974 (Desrosiers et al., 1974). Accumulating studies have uncovered the dynamic and delicate interplays between m^6^A modification and cancer development (Chen et al., 2019; Han et al., 2019; Liu et al., 2018). In colorectal cancer, the prognostic value of the m^6^A RNA methylation regulators YTHDF1 and HNRNPC has been described (Zaccara et al., 2019). However, little is known about the role of m^6^A regulators in the progression and prognosis of RC. In the present study, we analyzed the m^6^A modification system and constructed a comprehensive model for the prognostic prediction of RC. The m^6^A methylation regulators were significantly dysregulated in RC tumor tissues compared with normal tissues based on the TCGA-READ transcriptome profiling data (Figure 1), strongly indicating that these m^6^A regulators may play a key role in RC.

Our analysis also revealed that downregulation of YTHDC2 and METTL14 is associated with an unfavorable prognosis for RC patients. YTHDC2 was shown to have the tumor inhibitory effect on head and neck squamous cell carcinoma (HNSCC) and esophageal squamous cell carcinoma (ESCC) (Yang et al., 2020; Zhao and Cui, 2019; Zhou et al., 2020). Depletion of YTHDC2 significantly prompted cancer cell growth via regulating several important cancer-related pathways, including p53, NF-κB, and JAK–STAT signaling pathways (Yang et al., 2020). These findings together with our results demonstrated a tumor suppressive role of YTHDC2 in cancer. On the contrary, it has been reported that YTHDC2 can act as an oncogenic protein in pancreatic cancer (Fanale et al., 2014). Tanabe et al. also found that YTHDC2 promotes metastasis of colon cancer by bolstering Twist1 and HIF-1α translation (Tanabe et al., 2016). METTL14 was shown to exert its oncogenic role by regulating MYB and MYC through m^6^A modification in acute myeloid leukemia (AML) (Weng et al., 2018), whereas it also suppressed tumorigenesis and metastasis of bladder cancer or hepatocellular carcinoma (HCC) (Gu et al., 2019; Ma et al., 2017). Importantly, our findings were partially consistent with the previous study showing that downregulation of METTL14 was associated with poor clinical outcomes of CRC (Liu T. et al., 2019). These seemingly contradictory observations reflect the complexity of the m^6^A regulatory network in cancer, and suggest that the m^6^A regulators may possess distinct functions in the context of different cancer.

To evaluate the clinical significance of the m^6^A regulators, we constructed a prognostic model for RC survival by incorporating the expression of YTHDC2 and METTL14 through the LASSO Cox regression (Figure 1). The risk score of the patient was calculated by incorporating YTHDC2 and METTL14 expression into a risk signature, and RC patients from the TCGA dataset were then stratified into the low-risk and high-risk groups (Figure 2). The survival analysis showed that the high-risk group has significantly shorter overall survival and free relapse survival time compared with the low-risk group. Convincingly, the prognostic value of the model was further validated in RC patients from the GEO dataset (Figure 3). Moreover, the pathways associated with tumor progression were investigated (Figure 4), which provides a potential molecular basis for YTHDC2 and METTL14 mediated RC development. Hence, this novel prognostic model may bring a new view of angle for precise prediction of survival in RC.

## Conclusion

The aberrant expression of the m^6^A RNA methylation regulators, particularly YTHDC2 and METTL14, was significantly associated with the prognosis of RC. Our study proposed for the first time a risk signature incorporating these two m^6^A regulators, which might also be greatly beneficial for the future development of optimal therapies in RC.

## Data Availability Statement

The datasets presented in this study can be found in online repositories. The names of the repository/repositories and accession number(s) can be found in the article/Supplementary Material.

## Author Contributions

YC and SW conducted the study and analyzed the data. WC provided critical comments and edited the manuscript. XZ designed the study. XZ and ZZ analyzed the data and wrote the manuscript. All authors contributed to the article and approved the submitted version.

## Conflict of Interest

The authors declare that the research was conducted in the absence of any commercial or financial relationships that could be construed as a potential conflict of interest.
