# Supplementary material for: Prognostic Implication of the m6A RNA Methylation Regulators in Rectal Cancer
Source: Front Genet. 2021 Jun 3;12:604229. doi: 10.3389/fgene.2021.604229 (PMC8209494; doi:10.3389/fgene.2021.604229)
Supplement: Supplementary file 1 [file Data_Sheet_1.PDF]

Prognostic implication of the m^6A RNA methylation regulators in rectal cancer

Yajie Chen [1,2,#], Shanshan Wang [2,3,#], William C. Cho [4], Xiang Zhou [2,3,5,*], and Zhen Zhang [1,2,*]

[1] Department of Radiation Oncology, Fudan University Shanghai Cancer Center, Fudan University, Shanghai 200032, China

[2] Department of Oncology, Shanghai Medical College, Fudan University, Shanghai 200032, China

[3] Fudan University Shanghai Cancer Center and Institutes of Biomedical Sciences, Fudan University, Shanghai 200032, China

[4] Department of Clinical Oncology, Queen Elizabeth Hospital, Hong Kong, China

[5] Key Laboratory of Breast Cancer in Shanghai, Fudan University Shanghai Cancer Center, Fudan University, Shanghai, China

Running Title: The prognostic value of m^6A regulators in RC

[#] Equal contribution.

[*] Corresponding Authors:

Zhen Zhang, Department of Radiation Oncology, Fudan University Shanghai Cancer Center, Shanghai 200032, P. R. China. Email: zhen_zhang@fudan.edu.cn

Xiang Zhou, Fudan University Shanghai Cancer Center and Institutes of Biomedical Sciences, Fudan University, Shanghai 200032, P. R. China. Email: xiangzhou@fudan.edu.cn

Supplementary figures

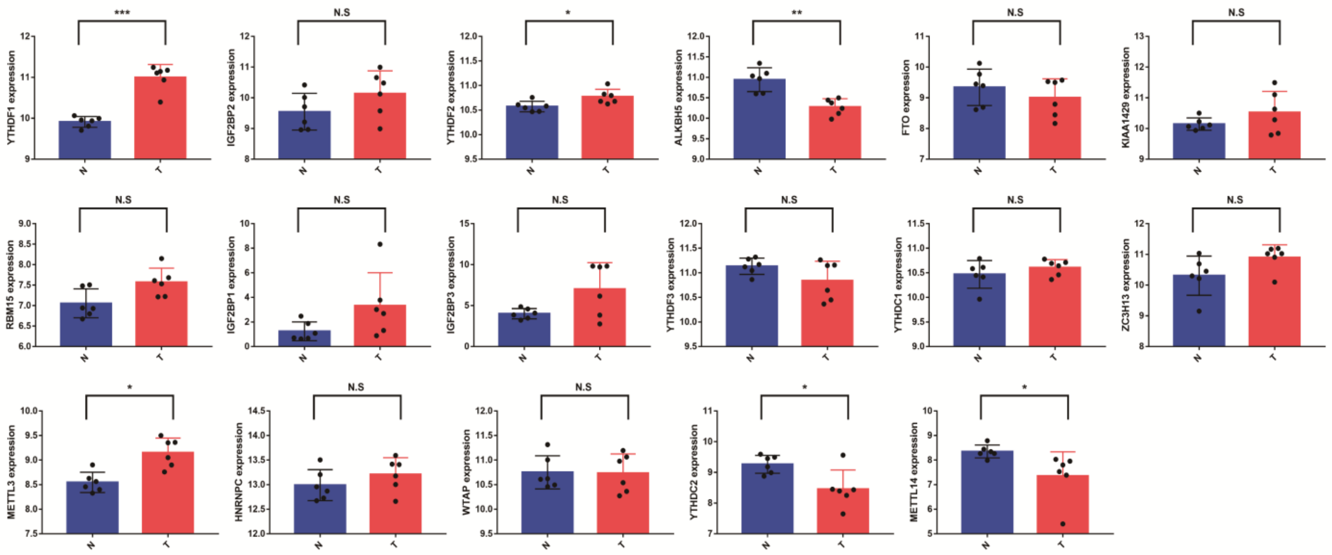

Supplementary Fig. 1 The expression of the 17 m^{6}A regulator genes in the 6 paired rectal tumors and normal rectal samples (paired T-tests). *P < 0.05, **P < 0.01, and ***P< 0.001.

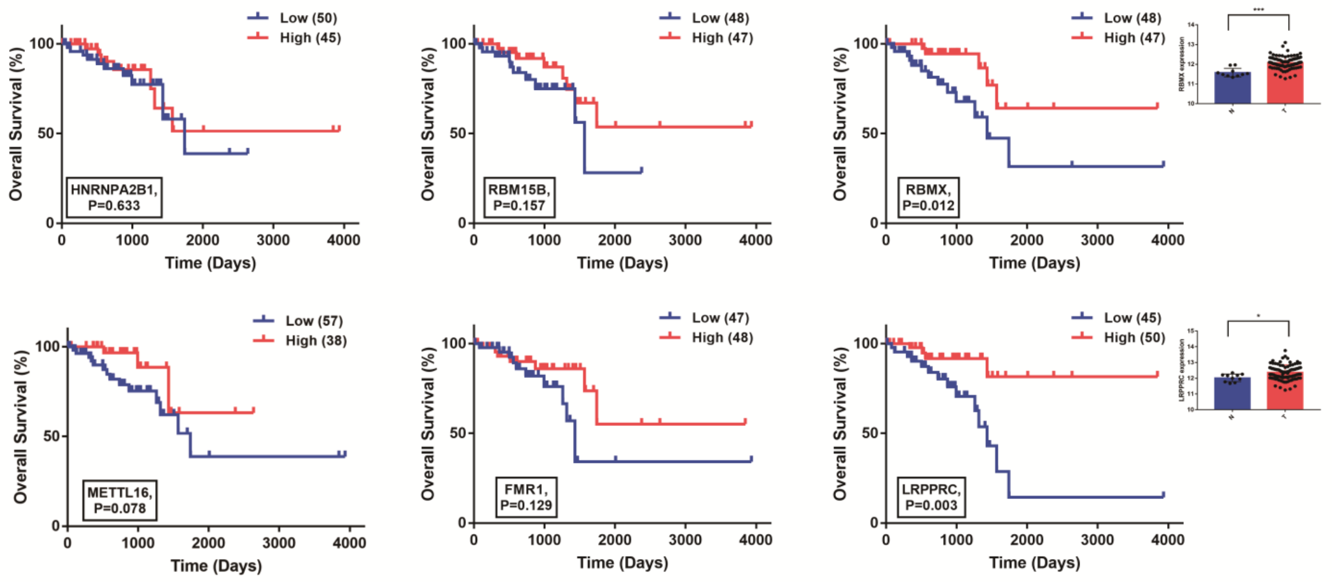

Supplementary Fig. 2 Comparison of overall survival with differential expression of the 6 m^{6}A regulators: HNRNPA2B1, RBM15B, RBMX, METTL16, FMR1, and LRPPRC.
